# Primary melanoma of the prostate: case report and review of the literature

**DOI:** 10.1186/s12894-015-0052-3

**Published:** 2015-07-14

**Authors:** Georgi Tosev, Timur H. Kuru, Johannes Huber, Gerald Freier, Frank Bergmann, Jessica C. Hassel, Sascha A. Pahernik, Markus Hohenfellner, Boris A. Hadaschik

**Affiliations:** Department of Urology, University of Heidelberg, Im Neuenheimer Feld 110, D-69120 Heidelberg, Germany; Institute of Pathology, University of Heidelberg, Heidelberg, Germany; Department of Dermatology, University of Heidelberg, Heidelberg, Germany

**Keywords:** Prostate, Prostatic neoplasm, Prostatectomy, Ipilimumab, Nivolumab

## Abstract

**Background:**

Primary melanoma of the prostate has an extremely rare incidence. Only five cases have been reported in the literature and prognosis is poor. The most likely origin of prostatic melanoma is the transitional epithelium of the prostatic urethra. Surgical care for primary melanoma of mucosal sites is less well established than for primary cutaneous melanoma, but excision of the primary is recommended if the patient has no systemic disease.

**Case presentation:**

Here, we describe a case of primary malignant melanoma of the prostate. A 37-year-old male patient with history of both chemo- and radiation therapy for Hodgkin’s disease was admitted to the University Hospital Heidelberg on suspicion of pleomorphic sarcoma of the bladder. In-house diagnostic work-up revealed a malignant melanoma of the prostate. We then performed radical prostatectomy with extended lymphadenectomy. Despite presumably curative surgery, the patient suffered from early relapse of disease with pulmonary metastasis. Systemic chemotherapy and subsequent immuno-oncologic treatment was thereafter initiated.

**Conclusion:**

Since prostatic melanoma is a rare disease and a melanoma metastasis of unknown primary is the differential diagnosis, a multidisciplinary approach including early imaging to rule out possible metastases and to search for another potentially existing primary is advisable. To prevent complications related to local tumor progression and to receive tissue for mutational analysis, we recommend complete surgical resection to reduce the tumor mass. Novel immune and targeted oncologic therapies can lead to an improved survival in some cases and support of clinical trials is needed.

## Background

Primary melanoma of the prostate has an extremely rare incidence. The first report of this disease was in 1980. Since then only five cases have been reported world-wide [1–5]. We report on one case including diagnosis, surgical management, and subsequent therapy with darcabazine, ipilimumab and nivolumab.

## Case presentation

A 37-year old man was admitted to the University Hospital Heidelberg for a second opinion after diagnosis of a pleomorphic sarcoma of the bladder in April 2012. His medical history included Hodgkin’s disease stage IIb which was first diagnosed in August 2004 in supraclavicular and mediastinal lymph nodes. In 2004 he received two cycles each of BEACOPP and ABVD chemotherapy, resulting in complete remission. Consolidating treatment consisted of involved-field radiotherapy with 30 Gy in 2005.

The patient reported that one month before his first consultation at our Clinic he suffered from macrohaematuria and urinary retention. He was admitted to an outside urology unit and a transurethral resection was performed. Histopathology reported a pleomorphic sarcoma of the bladder neck with malignant melanoma as a possible differential diagnosis.

Neither digital rectal examination, nor a serum prostate antigen of 1.57 ng/ml, were indicative of primary prostate cancer, but in-house cystoscopy showed an asymmetric prostate enlargement with purple discoloration. Thus, transrectal prostate biopsy was performed with inconclusive results, followed by a diagnostic TUR-P yielding two specimens from the dubious area of the left prostate lobe above the level of the verumontanum. Final histopathology and immunohistochemical staining showed positive expression of Vimentin, Melan A, CD56, and HMB 45 and negative expression of CD30, Desmin, PSMA, S100, AE 1/3, CD34, LCA, PLAP, BerEp4, EMA, CD68, Inhibin and Calretinin, which confirmed the diagnosis of a melanoma [6]. Clear cell sarcoma was excluded via the lack of EWSR-ATF-1 translocation in the areas of the lesion analyzed.

**Fig. 1:**
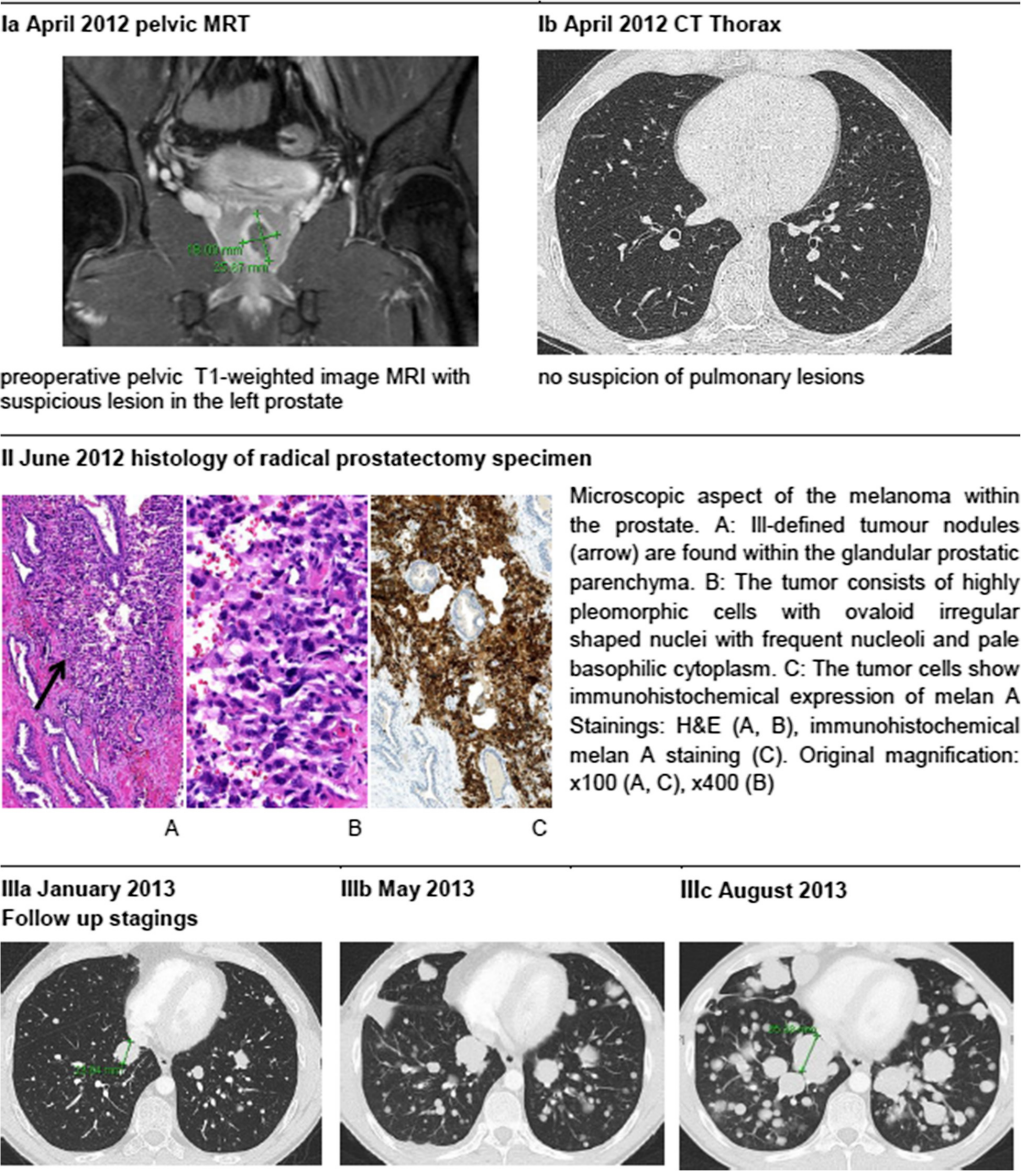
Case time-line

No evidence for primary melanoma at other sites, particulary skin and mucosal by dermatologic, ophthalmologic and otolaryngologic examinations, as well as colonoscopy and gastroscopy, or distant metastases were found on imaging (CT with contrast of chest and abdomen and pelvic MRI with contrast in April 2012; Fig. 1, Ia and Ib). Therefore, open retropubic radical prostatectomy with extended lymph-node dissection was performed with curative intention in June 2012. Histopathologic examination (Fig.1, IIa, IIb, IIc) revealed a primary melanoma of the prostate with clear surgical margins (pR0) and tumour-free lymph nodes (pN0 0/22). One year after surgery the patient was completely continent and reported normal erectile function. Additionally, follow-up imaging did not show any local recurrence in the small pelvis. However CT imaging of the chest and, abdomen, and an MRI of the head with contrast in October 2012 and January 2013 (Fig. 1, IIIa) detected multiple small pulmonary lesions of up to 24 mm in size, suspicious for pulmonary metastases. Bronchoscopy was performed and biopsies from the pulmonary nodules were taken. Histopathology showed metastases of the melanoma confirming systemic relapse. According to the results of subsequent molecular analyses, no BRAF, cKIT, NRAS or GNAQ mutations and again no EWSR-1-translocation were identified. Systemic therapy with DTIC (Dacarbazine) 1000mg/m^2^ for three cycles was initiated. Due to disease progression (Fig. 1, IIIb), therapy was switched to Ipilimumab (3 mg/kg). Staging after four cycles of Ipilimumab again revealed a massive disease progression and the patient was subsequently included in a phase three study of an anti-PD1 antibody receiving five cycles (NCT 01721746). As before, the patient experienced a pronounced progression of lung and hilar lymph node metastases (Fig. 1, IIIc). Another treatment regimen consisting of chemotherapy with carboplatin and paclitaxel was offered to the patient, but was declined. The patient died 16 months after initial diagnosis in palliative care.

### Consent

Written informed consent was obtained from the parents of the patient for publication of this Case report and any accompanying images. A copy of the written consent is available for review by the Editor of this journal.

## Discussion

Primary melanoma of the prostate is exceedingly rare. In the present case our patient suffered from melanoma of the prostate as a secondary malignancy.

The patient’s history of Hodgkin disease (HD) and prior treatment with chemotherapy and radiochemotherapy (RT) may play a pathogenetic role in the development of the secondary malignancy. HD often involves cervical and mediastinal lymph nodes, and RT is known to target these nodal regions resulting in the irradiation of adjacent mammary tissue and lung. The risk of secondary lung cancer, breast, bladder, gastric and oesophageal cancer has been associated with radiation dose and with field size, but it is mostly described > 10 years after treatment [7, 8]. Similarly, secondary malignancy rates after BEACOPP-chemotherapy are 5.7–6 %, respectively [9]. Among adult survivors of childhood cancer, the prevalence of adverse health outcomes is high and an ongoing health monitoring for this group of patients is very important [10]. Due to the uncommon localization, no previous staging CT or MRI included the prostate in the present case. Only symptoms of locally advanced disease such as hematuria and urinary retention lead to further diagnostic work-up and to the final diagnosis. As seen on upon MRI analysis, the location of the tumor was in the transition zone of the prostate. Thus, the most likely origin of the prostatic melanoma was the transitional epithelium of the prostatic urethra. However, we cannot completely exclude that the patient had a stage IV melanoma with unknown primary where the prostate was the first location of metastases. In disseminated metastatic melanoma it has been reported that the prostate may be involved in 3 % of autopsies.

Initial management of cutaneous melanoma after biopsy is directed by depth of tumor, presence of ulceration, and dermal mitoses and may involve wide local excision with margins ranging from 1 to 2 cm plus/minus sentinel lymph node biopsy, and adjuvant interferon or immuno-oncologic therapy depending on final staging [11]. Surgical care for primary melanoma of mucosal sites is less well established than for primary cutaneous melanoma, but excision of the primary is always the goal if the patient has no systemic disease.

Despite low chances of a cure, we performed radical prostatectomy (RP) with extended lymph node dissection due to clinical lower urinary tract symptoms and to prevent further local complications. RP is a strong independent predictor of survival benefit in patients with prostate cancer [12]. However, radical prostatectomy can also have detrimental side effects, especially if performed at low volume hospitals [13]. Thus, the decision of dissection of the tumor was an individual approach, based on the current knowledge of the treatment of prostate carcinoma and melanoma and based on the fact that macrohaematuria and lower urinary tract symptoms significantly impair quality of life. In the present case, the patient fully recovered lower urinary tract function after surgery.

Unfortunately, the patient suffered from systemic relapse with pulmonary metastases shortly afterwards. Systemic therapy was initiated, and 16 months after initial diagnosis the patient died due to multi-organ failure. Advanced imaging such as PET/CT may have detected systemic disease earlier than the staging we had performed. However, first metastases occurred in the lungs for which PET scanning is not clearly superior to CT. In addition, the management of the patient would not have changed significantly since one of the main indications for surgery was to improve micturition and to prevent local complications.

Systemic therapeutic options for advanced melanoma have substantially increased over the last several years. They can be divided into targeted therapies such as BRAF, MEK, cKIT inhibitors or immuno-oncologic treatments such as CTLA4 and PD-(L)-1 antibodies which manipulate the immune system. At present, trials are addressing various combination therapies to improve overall survival.

Melanomas arising in visceral organs are less likely to be BRAF or NRAS positive. While mucosal melanomas are more likely to have cKIT mutations and if positive can benefit from targeted therapy such as tyrosine kinase inhibition, this was not the case in our patient. In the case report of Ma et al. before novel immunotherapeutic drugs were available, the patient survived three months on systemic treatment. Although our patient showed disease progression under therapy with novel immunotherapeutics, the overall survival was better than the case stated above. Whether this means that tumor growth was slowed down under treatment with checkpoint inhibitors is at this point speculative.

Interestingly, the anti-cytotoxic T lymphocyte-associated receptor 4 (CTLA4) antibody Ipilimumab is also under investigation for patients with metastatic, castration-resistant prostate cancer (mCRPC) [14].

## Conclusion

Due to the extreme rarity of prostatic melanoma, only very few cases have been reported. This increases the scientific importance of each individual case report. A multidisciplinary team approach to search for another potentially existing primary and early imaging to rule out possible metastases are advisable. In case of prostatic melanoma we recommend complete surgical resection with extended lymphadenectomy by experienced surgeons to prevent complications related to local tumor progression.

Advanced melanoma shows a wide heterogeneity and testing for different mutations of the tumor is needed to choose drug regimens individually for the patient. Novel immuno-oncologic therapy may lead to an improved survival in some cases.

## Abbreviations

BEACOPP: Bleomycin Etoposide, Adriamycin (doxorubicin), Cyclophosphamide, Oncovin = Vincristine, Procarbazine, Prednisone
ABVD: Adriamycin Bleomycin, Vinblastine, Dacarbazine
DTIC: Dacarbazine
RT: Radiotherapy
HD: Hodgkin disease
TUR-B: Transurethral resection of the bladder
TUR-P: Transurethral resection of the prostate
